# Behavioral discrimination and time-series phenotyping of birdsong performance

**DOI:** 10.1371/journal.pcbi.1008820

**Published:** 2021-04-08

**Authors:** Avishek Paul, Helen McLendon, Veronica Rally, Jon T. Sakata, Sarah C. Woolley

**Affiliations:** 1 Dept. Electrical & Computer Engineering, McGill University, Montreal, Canada; 2 Dept. Biology, McGill University, Montreal, Canada; 3 Keck Center for Integrative Neuroscience, University of California, San Francisco, San Francisco, California, United States of America; 4 Centre for Research on Brain, Language, and Music, McGill University, Montreal, Canada; University of California at Berkeley, UNITED STATES

## Abstract

Variation in the acoustic structure of vocal signals is important to communicate social information. However, relatively little is known about the features that receivers extract to decipher relevant social information. Here, we took an expansive, bottom-up approach to delineate the feature space that could be important for processing social information in zebra finch song. Using operant techniques, we discovered that female zebra finches can consistently discriminate brief song phrases (“motifs”) from different social contexts. We then applied machine learning algorithms to classify motifs based on thousands of time-series features and to uncover acoustic features for motif discrimination. In addition to highlighting classic acoustic features, the resulting algorithm revealed novel features for song discrimination, for example, measures of time irreversibility (i.e., the degree to which the statistical properties of the actual and time-reversed signal differ). Moreover, the algorithm accurately predicted female performance on individual motif exemplars. These data underscore and expand the promise of broad time-series phenotyping to acoustic analyses and social decision-making.

## Author summary

Variation in vocal performance can provide information about the social context and motivation of the signaler. For example, prosodic changes to the pitch, tempo, or loudness of speech can reveal a speaker’s emotional state or motivation, even when speech content is unchanged. Similarly, in songbirds like the zebra finch, males produce songs with the same acoustic elements but with different vocal performance when courting females and when singing alone, and females strongly prefer to hear the courtship song. Here we integrated behavioral and computational approaches to reveal the acoustic features that female zebra finches might use for social discrimination. We first discovered that females excelled at distinguishing between brief phrases of courtship and non-courtship song. We next extracted thousands of time-series features from courtship and non-courtship phrases using a highly-comparative time series analysis (HCTSA) toolbox, and trained machine learning algorithms to use those features to discriminate between phrases. The machine learning algorithm identified features important for discriminating between courtship and non-courtship song phrases, some of which have not been implicated before, and reliably predicted the discrimination abilities of females. Together, these data highlight the power of expansive and bottom-up approaches to reveal acoustic features important for social discrimination.

## Introduction

A wide range of animals rely on acoustic signals for social communication (reviewed in [1,2]). Receivers decode and use “information” in the vocalizations of signalers to shape their own behavioral responses. For example, the acoustic content of vocalizations can provide insight into the presence and type of predators or food sources near to a signaler, as well as information about the signaler’s species and identity [3,4]. In addition to variation in the content of vocal signals, the manner in which a particular vocalization is produced, including variation in prosodic features such as pitch, tempo, or rhythm, also provide important social and contextual information (reviewed in [5–7]). Indeed, subtle modifications in the performance of a fixed set of vocalizations by songbirds and humans can provide cues to the arousal, reproductive state, or social motivation of the signaler [6,8–10]. However, uncovering the acoustic modifications most important to receivers remains a challenge.

Songbirds like the zebra finch offer an excellent opportunity to investigate acoustic features for auditory processing and discrimination [1,11,12]. Female zebra finches use the songs of male zebra finches to identify individuals and to select mates [13–15]. While decades of research have investigated the production and perception of song, much of the research has focused on a small subset of acoustic features, largely derived from studies in humans or other species that rely on acoustic signals for communication [16–18]. While this subset of acoustic features exhibits variation between individuals, between social contexts, and over development, it remains unclear whether they adequately capture the feature space important to female receivers.

Here, we took an expansive, bottom-up approach to delineate the feature space important for information processing by receivers. Zebra finches produce song bouts that consist of the repetition of a single, stereotyped sequence of acoustic elements (“syllables”) called a motif. Individual males produce the same motifs when singing to females during courtship interactions (“courtship” or “directed” motifs) and when singing alone (“non-courtship” or “undirected” motifs) but song performance differs in a variety of ways across these social contexts, presumably as a way for males to increase the attractiveness of their songs [19–21]. Consistent with this notion, females prefer to listen to bouts of courtship song over bouts of non-courtship song [22,23]. Because bouts of courtship and non-courtship song consist of motifs with the same syllables organized in the same sequence, this finding suggests that changes to the acoustic structure of motifs could have reproductive consequences. While attempts have been made to discern the acoustic features important for this discrimination and preference, to date only a few, hand-picked features have been explored (e.g., fundamental frequency and spectral entropy of syllables: [22–24]). In addition, studies to date often emphasize acoustic features that rely on comparisons between multiple renditions of motifs [22,23], and the degree to which females are able to detect context-dependent variation within single renditions of motifs remains unclear. To gain deeper insight into the acoustic basis of social discrimination, we employed operant techniques to examine the degree to which female zebra finches could differentiate between individual motifs of courtship and non-courtship songs. We then coupled novel and powerful feature extraction techniques with machine learning algorithms to elucidate the array of features that differentiate single motifs produced across different social contexts. Finally, we assessed the biological significance of the resulting algorithms by covarying algorithm performance with the behavioral performance of females.

## Results

### Female zebra finches can classify single motifs of courtship or non-courtship song

**Fig 1:**
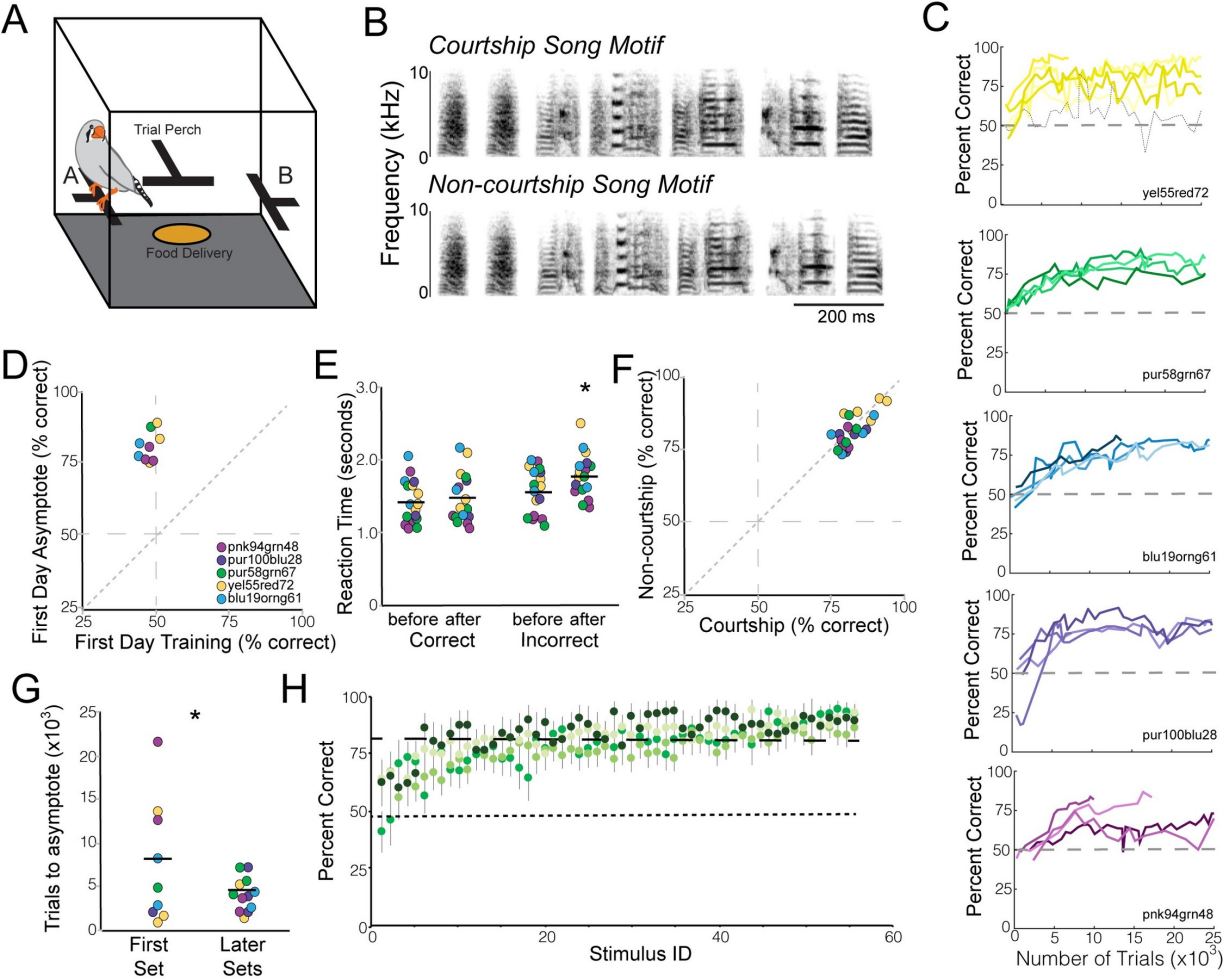
Female zebra finches can correctly classify single motifs as courtship or non-courtship motifs. (A) Diagram of the behavioral apparatus. Birds initiate song playback via the trial perch and classify the stimulus by hopping on perch A or B. Correct responses lead to brief access to food. (B) Examples of single courtship and non-courtship song motifs. The two notes at the beginning (introductory notes) are the same across all stimuli and provide no information for motif classification. (C) Performance (% correct) of individual females over the first 25,000 trials of training and testing on each of the five male stimulus sets. Colors indicate the different male stimulus sets, shades within a color are different females, dashed lines indicate 50% correct. The black line in the top panel depicts one bird who did not achieve asymptotic performance. (D) Females significantly improved their performance between the first day of training and the first day of asymptotic performance. Colors indicate the different male stimulus sets females were tested on, points are the mean performance of individual females on the stimulus sets. (E) Reaction times were significantly higher for incorrect responses after females reached asymptotic performance relative to during training and as compared to correct responses at either time point. (F) Females performed equally well on classifying both courtship and non-courtship stimuli (data after reaching asymptotic performance). Female performance across multiple stimulus sets is plotted. (G) Sequential testing of females on different stimulus sets indicates some savings on later stimulus sets. Specifically, females that were tested sequentially on stimulus sets from more than one male required fewer trials to reach asymptotic performance on later stimulus sets compared to their first stimulus set. (H) While female performance was generally high, females performed better on some individual motifs than others. Moreover, different females tended to perform similarly across stimuli. Plotted is the performance of four different females (indicated with different shades of green) on the 60 stimuli from a single male (30 courtship, 30 non-courtship; stimuli sorted based on average performance). The dashed at 76% indicates the average performance across all females and stimuli. Note that for some stimuli, all females perform worse than average (left side) and for others all females perform better than average (right side).

Female songbirds have been shown to prefer bouts of courtship song over bouts of non-courtship song, and variation in female preferences for courtship songs has been found to be linked with variation in the stereotypy of vocal performance across motif renditions [22,23]. However, the degree to which females attend to features present in a single motif rendition and can use these features for discrimination is unknown. Here, we tested the extent to which females differentiate between single motifs of courtship and non-courtship song by training female zebra finches (n = 11) to classify individual motif renditions as courtship or non-courtship motifs (two-alternative forced choice task; Fig 1A and 1B). We created five stimulus sets, each consisting of motifs from an individual male zebra finch, and trained and tested females on one stimulus set at a time. Some females (n = 6) were sequentially tested on up to three different male stimulus sets. Each stimulus set contained a matched number of courtship and non-courtship motifs (n = 23–38 motif exemplars per context per stimulus set) that were similar in their syllable composition and sequencing (Fig 1B).

Females (n = 9) learned to accurately classify single song motifs from a male as courtship or non-courtship motifs. Females initially performed at chance levels (50% accuracy) but gradually improved their ability to correctly classify individual motifs and maintained that level of performance thereafter (“asymptotic performance”; see Methods; Fig 1C). Across females, performance significantly improved between the first day of training and the first day of asymptotic performance (mixed-effects model; F_(1,12)_ = 110.51; p<0.0001; Fig 1D). Reaction times also changed over the course of training. In particular, at the start of training, females had similar reaction times when making correct and incorrect responses. However, after females reached asymptotic performance, reaction times were significantly longer for incorrect responses than for correct responses (mixed-effects model; F_(1,28)_ = 4.86; p = 0.0357; Fig 1E). On average, females reached asymptotic performance within 14.5±2.2 (mean±SEM) days of training (range: 5–35 days). Females performed an average of 860±63 trials/day (range: 50–3495 trials) and required an average of 12.7±2.3 thousand trials to reach asymptotic performance. After reaching asymptotic performance, females correctly classified 81.9±0.2% (range: 76–93%) of motifs (Fig 1D).

Females that were sequentially tested on stimulus sets from different males showed faster behavioral improvements on later stimulus sets compared to their first stimulus set. In particular, females required significantly fewer trials on later test sets to reach asymptotic performance (mixed-effects model; F_(1,29)_ = 44.19; p<0.0001; Fig 1G). Whether this difference indicates that song features important for the song discrimination are shared across males or demonstrates the effects of practice on task performance is unclear.

Classification ability was not restricted to songs from a single context or the songs of a single male. Females performed equally well on courtship and non-courtship stimuli. In particular, asymptotic performances on courtship (82.2±1.4% correct) and non-courtship stimuli (80.5±1.4% correct) were not significantly different (mixed-effects model; F_(1,20.34)_ = 2.20; p = 0.1535; Fig 1F), suggesting that females did not use a response bias (e.g., hop left) to solve the task but were able to properly categorize both courtship and non-courtship motifs. Females also performed above chance regardless of the male stimulus set. We trained different females with stimulus sets from five different males (Fig 1C). We found that while there was variation in female performance across the stimulus sets (mixed-effects model; F_(4,25.02)_ = 3.03; p = 0.0364), overall, females performed significantly above chance on stimuli from all five males (t-test; p<0.0001 for all stimulus sets; Fig 1D). Moreover, the ability to discriminate courtship and non-courtship motifs regardless of male song was also evident in females sequentially tested on stimulus sets from different males: females that were able to discriminate the courtship and non-courtship motifs of one male were similarly able to discriminate the motifs of another male.

Not only did females make categorical distinctions between courtship and non-courtship motifs, but there was substantial similarity among females in their performance on individual stimuli. For example, as shown in Fig 1H, all females tested on the songs of one male (“pnk94grn48”) tended to have lower than average performance on certain stimuli and higher than average performance on others. Overall, females covaried in their performance on training stimuli, with significant and positive correlations between females for 35 of 38 comparisons (see Methods).

### Female zebra finches can generalize to novel song renditions

A female bird could use at least two different strategies to categorize courtship and non-courtship stimuli. One approach would be to simply memorize the rewarded response for each motif exemplar in the training set. Alternatively, females could use the acoustic properties that distinguish the stimuli to construct categories and assign stimuli accordingly. In order to determine which strategy the females used, we tested the responses of each female to novel exemplars of a male’s courtship and non-courtship motifs that were not included in the original training sets. These ‘probe trials’, administered after females reached asymptotic performance, were randomly interspersed between training trials at a low rate of 10–20% and were rewarded at a fixed rate regardless of which response perch the female chose (see Methods).

**Fig 2:**
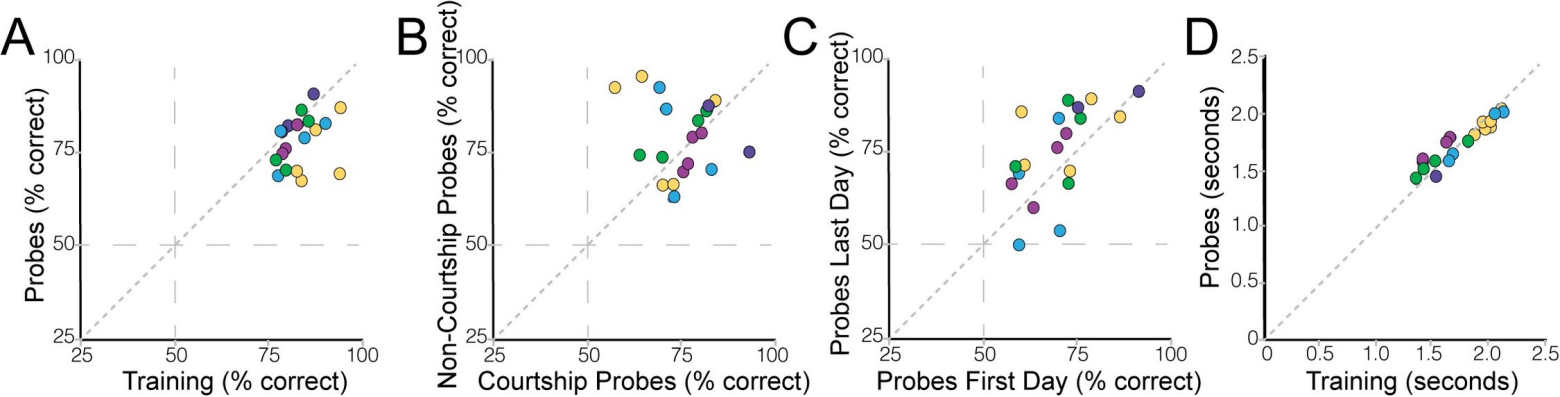
Female zebra finches can generalize classification to novel probe stimuli. (A) Females perform significantly above chance in classifying novel probe stimuli. However, females are slightly but significantly better at classifying training stimuli, indicating that familiarity improves classification accuracy. There were no significant differences in performance between courtship and non-courtship probes (B) or between the first and last day of testing on probe stimuli (C). (D) Similarly, reaction times were not significantly different between training and probe stimuli.

Consistent with the notion that females create broad categories to differentiate between courtship and non-courtship motifs, females were able to accurately categorize the probe stimuli. As illustrated in Fig 2A, females correctly classified probes on 77±2% of tests. In particular, they performed significantly above chance (50%) on probe motifs for all stimulus sets (t-tests; p<0.001 for each). Moreover, there was no difference in performance between courtship and non-courtship probes (F_(2,21.5)_ = 0.71; p = 0.4100; Fig 2B), and successful discrimination of probe stimuli was not different between the first and last day of testing (F_(1,20.7)_ = 3.02; p = 0.0971; Fig 2C). Furthermore, reaction times for the probes were not significantly different from the reaction times to training stimuli (F_(1,59.2)_ = 1.96; p = 0.1670; Fig 2D), implying that the randomly interspersed probe trials were seamlessly integrated into the ongoing task structure. However, females were slightly but significantly better at classifying the training stimuli than the probe stimuli (F_(1,54.5)_ = 7.10; p = 0.0101; Fig 2A), suggesting that familiarity with the stimulus improved accuracy.

### Combining highly comparative time-series analyses (HCTSA) with machine learning algorithms to discriminate between courtship and non-courtship motifs

Given that females readily distinguish courtship motifs from non-courtship motifs, we next sought to identify features that would be available to receivers for song discrimination. To this end, we took an expansive, bottom-up approach, combining a novel and powerful time-series analysis toolbox with machine learning algorithms. Individual song motifs are brief (~500 ms) and complex acoustic sequences that can be summarized using a variety of time-series metrics (e.g., power spectrum, autocorrelation, etc.). While most studies to date have analyzed a handful of pre-determined acoustic features to describe and quantify birdsong, the degree to which these features sufficiently describe song or capture the features important to receivers remains unclear. Highly comparative time-series analyses (HCTSA) is a novel toolbox that combines roughly one thousand distinct, interdisciplinary time-series analysis techniques (“operations”) to compute thousands of individual “features” of time series data [25,26]. For example, one operation computes the autocorrelation of a time series. The autocorrelation is computed at multiple different lags, and the output for each lag constitutes a different feature. Features computed in the HCTSA extraction include various summary statistics (e.g., mean, median, variance) of time-series operations (e.g., power spectral measures, autocorrelation functions, information theoretic and entropy measures, physical non-linear time-series analyses). Such features (e.g., variance of the power spectral density of a time series, 1^st^-order autocorrelation) have been used in conjunction with machine learning algorithms to analyze and differentiate diverse types of time series data, including ethograms in fruit flies and neurophysiological and acoustic measures of speech in humans [25]. We adopted a similar approach, coupling HCTSA feature extraction with machine learning, to reveal acoustic features that could be used by receivers for social discrimination.

**Fig 3:**
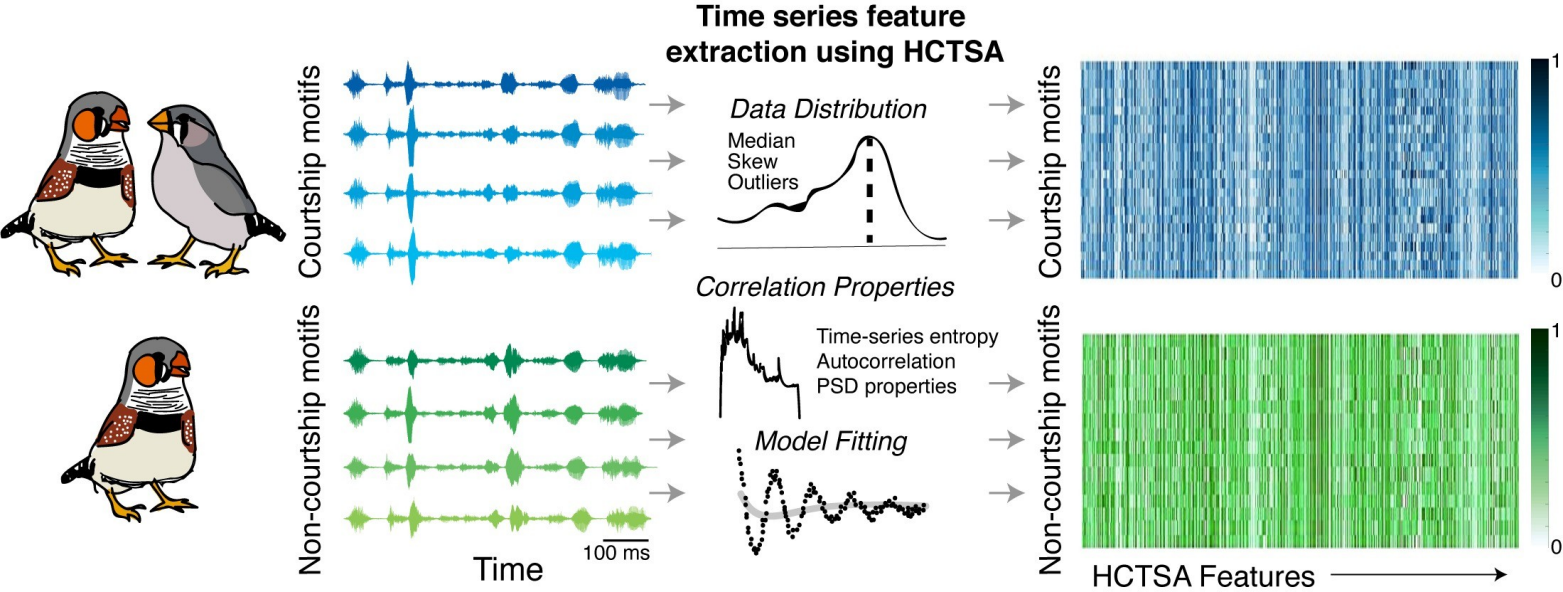
Analysis pipeline for HCTSA feature extraction followed by classification using machine learning algorithms. We analyzed the acoustic structure of courtship (top; blue) and non-courtship (bottom; green) motifs produced by male zebra finches. Next to the cartoons of zebra finches are the acoustic waveforms of four renditions of a motif produced by one male zebra finch. The same sequence of acoustic elements is performed during courtship and non-courtship song. Using numerous time-series “operations” (e.g., autocorrelation, power spectral density (PSD)), the HCTSA toolbox consistently extracted 5525 “features” from each individual waveform (an example of a “feature” is the median of the PSD computed using a Fourier transform). We created a matrix of the extracted features for all courtship (blue) and non-courtship (green) motifs. Plotted in the right column are heatmaps of normalized values (see Methods), with each row representing a single motif and the color in each column representing the normalized value of an acoustic feature. Darker colors indicate values closer to 1, lighter colors indicate values closer to 0.

Thousands of acoustic features from the courtship and non-courtship motifs of 17 male zebra finches were extracted using HCTSA (Fig 3). While the HCTSA toolbox can extract >7800 features for each motif, the precise number of features extracted for each data set depends on the specifics of the data, and features are filtered out if, for example, they are constant or not appropriate for the data (e.g., methods for summarizing a positive-only distribution are filtered out if the data set contains negative values). For our dataset, 5525 features were consistently extracted for each individual courtship motif (33.8±4.2; mean±SEM motifs per male; range: 14–85 motifs) and non-courtship motif (29.5±4.0 motifs per male; range: 6–68 motifs), and these data were subsequently run through bagged decision trees (BGDT), a type of multivariate classification algorithm.

**Fig 4:**
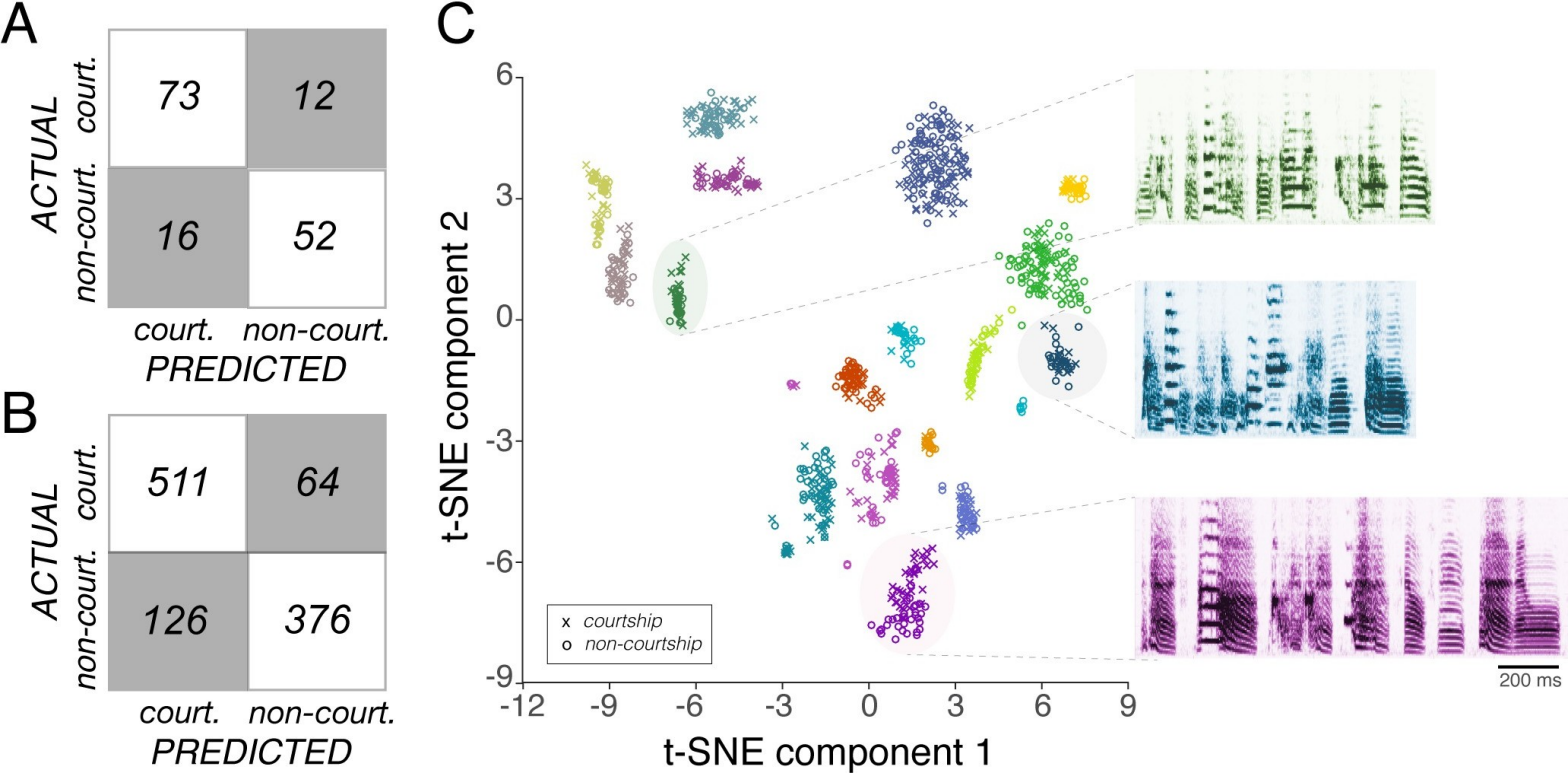
Performance of bagged decision trees (BGDTs) classifying exemplars of courtship and non-courtship motifs. Plotted are confusion matrices that indicate the actual (y-axis) categories (courtship vs. non-courtship) and predicted categories generated by the BGDTs (x-axis) for (A) an individual classifier (male ‘prpred’; 81.7% overall accuracy) and (B) the combined classifier (82.4% overall accuracy). Classification accuracy of the individual or combined classifiers did not significantly correlate with the sample size of the dataset (S1 Fig). (C) Data visualization of courtship and non-courtship motifs using t-distributed stochastic neighbor embedding (tSNE), a non-linear technique for dimensionality reduction (different symbols within each colored cluster; x is a courtship motif, o is a non-courtship motif). Shown are example spectrograms of non-courtship motifs from three different birds.

We first used BGDTs to classify motifs from each individual bird (n = 17) as courtship or non-courtship (“individual classifiers”). This gave us 17 different classifiers, one for every male, that could each accurately categorize the motifs of an individual bird as courtship or non-courtship based on the 5525 features extracted using HCTSA. An example of the performance accuracy (% correct) of an individual classifier (male ‘prpred’) is shown in Fig 4A. For this bird’s song, the individual classifier accurately categorized 81.7% of the motifs. Across the 17 individual classifiers the average performance accuracy (see Methods) was 85.1±3.1%, and classification accuracy of each individual classifier did not significantly correlate with the sample size of their respective datasets (Panel A in S1 Fig).

While individual classifiers were able to accurately classify the courtship and non-courtship motifs of the birds they were trained on, they did not generalize well to the courtship and non-courtship motifs of other birds. For example, the individual classifier for male prpred (shown in Fig 4A) accurately classified 81.7% of prpred’s motifs but only 48.4±2.5% (range: 35–70%) of the motifs of other males. This is not surprising given the wide range of acoustic properties across birds’ songs compared to the minimal variation between the courtship and non-courtship motifs from the same bird (Fig 4C). To address this, we next trained a classifier to simultaneously classify the songs from all 17 males (“combined classifier”; see Methods). Despite the diversity of acoustic structures across birds, the combined classifier performed almost as well as the individual classifiers, correctly categorizing 82.4% of the motifs (Fig 4B). These analyses indicate that there is consistent information in the acoustic waveforms of song motifs to reliably differentiate between the same sequence of syllables performed in different social contexts.

“Importance scores” generated by BGDT classifiers reflect the contribution of individual features to classification. Based on the distributions of importance scores for the combined classifier (S2 Fig), we chose to examine the 50 features with the highest importance scores (S1 Table). We found that the top 50 features were clustered among a handful of operations, suggesting that these operations may be especially useful in discriminating courtship and non-courtship motifs (Table 1). The most represented operations included calculations of a power spectral density (PSD; e.g., using the operation “SP_summaries”; 8 features), time irreversibility (12 features), and autoregressive model fitting (6 features). Some of these features are outputs from operations that have been previously used to quantify the acoustic properties of song (e.g., PSDs) whereas other prominent operations, such as time irreversibility statistics, are novel with regard to distinguishing courtship and non-courtship song in zebra finches and in quantifying acoustic properties of birdsong in general.

**Table 1:** Summary of operations for the top 50 features (based on importance scores).

Operation Type	Operation Name (as per HCTSA)	Number of occurrences (counts)	Description
Time Irreversibility Statistic	CO_trev	8	Normalized nonlinear autocorrelation
	DK_timerev	4	Time reversal asymmetry statistic
Spectral Operations	SP_summaries	8	Power spectral densities (PSDs) computed using different transformations (welch, fourier, or periodogram) with different window types (hamming or rectangular)
Multi-level discretization of time series and local pattern Identification	SB_MotifThree	1	Motifs of 3 pulled from a coarse-graining of time series into discrete alphabets
	SB_BinaryStats	1	Statistics on binary symbolization of time series
	SB_TransitionMatrix	2	Transition probabilities between different time series states
	SB_TransitionAlphabet	3	Changes to transition probabilities that occur with changes in alphabet size
Statistics of auto-regressive model fitting	MF_CompareAR	1	Comparison of model fits of various orders to a time series
	MF_arfit	3	Statistics of a fitted AR model (Schwartz’s Bayesian Criterion, Aikake’s Final Prediction Error, test residuals, confidence intervals, eigen decomposition)
	MF_CompareTest	1	Robustness of goodness of fit. Fits the model on the full time series and compares how well it predicts time series in different local time-series segments.
	MF_steps_ahead	1	Variation in goodness of model predictions across a range of prediction lengths
Stationarity Measures	SY_spreadRandomLocal	3	Bootstrap-based stationarity measure. Summarizes how mean, standard deviation, skewness, and kurtosis vary in different local segments of the time series
	SY_localGlobal	1	Comparison of local statistics to global statistics of a time series (mean, standard deviation, median, interquartile range, skewness, kurtosis)
Permutation Entropy Metrics	EN_PermEn	2	Permutation entropy and multiscale entropy of a time series
Pre-processing comparisons	PH_walker	2	Simulation of a hypothetical walker moving in response to values of the time series at each point
	PP_iterrate	1	Pre-processing transformation iteratively applied to the time series
AutoMutual Information	CO_Histogram	1	Automutual information of the distribution using histograms
	CO_AddNoise	1	Changes in the automutual information with the addition of noise
Autocorrelation metrics	AC_33, AC_14	2	Autocorrelation of a time series at different lags
Heart Rate Variability	MD_hrv_classic	2	Classic heart rate variability statistic that describes non-linear fluctuations in time intervals
Local Forecasting	FC_LoopLocalSimple	1	Simple local forecasting as a function of window length
	FC_surprise	1	Quantification of "surprise" for a next data point given recent memory values.

To assess the utility of these 50 features for classification, we trained a BGDT classifier to classify motifs of all birds using only these top 50 features. Such a classifier correctly categorized 81.6% of the motifs, which is comparable to the performance of the classifier using all 5525 features (82.4%). In addition, the predictions generated by the classifier for each individual training stimulus using these top 50 features correlated with the classifications of females for those same stimuli (F_(1,283)_ = 26.6; p<0.001). BGDT classifiers that used a random selection of 50 features never performed as well as the BGDT classifier using the top 50 features (Monte Carlo simulations: 1000 iterations).

To provide comparison with other features commonly used to characterize birdsong, we also extracted song features with Sound Analysis Pro (SAP)[27]. We extracted 12 default features (mean and variance of mean frequency, of Wiener entropy, of pitch goodness, of frequency modulation, and of amplitude modulation, mean pitch, and mean amplitude) from the courtship and non-courtship motifs of all 17 males. We then trained a BGDT classifier to classify courtship and non-courtship motifs using these features, similar to our approach with the HCTSA combined classifier. Overall, the BGDT using SAP features accurately classified 74.7% of the motifs, which is lower than the accuracies of HCTSA-based classifiers using all features (82.4%) or using just the top 50 features (81.6%).

### Discrimination performance of the machine learning algorithm predicts discrimination performance of female birds

Both females and the combined classifier were able to accurately classify courtship and non-courtship motifs. In particular, the overall performance of the combined classifier was similar to the performance of female zebra finches. The combined classifier (Fig 4C) accurately classified 78% of the motifs from the five stimulus sets used for operant training, while females accurately classified around 82% of the training stimuli once they reached asymptotic performance (Fig 1D). These data raise the possibility that the algorithm could accurately model and predict female performance on individual stimuli.

**Fig 5:**
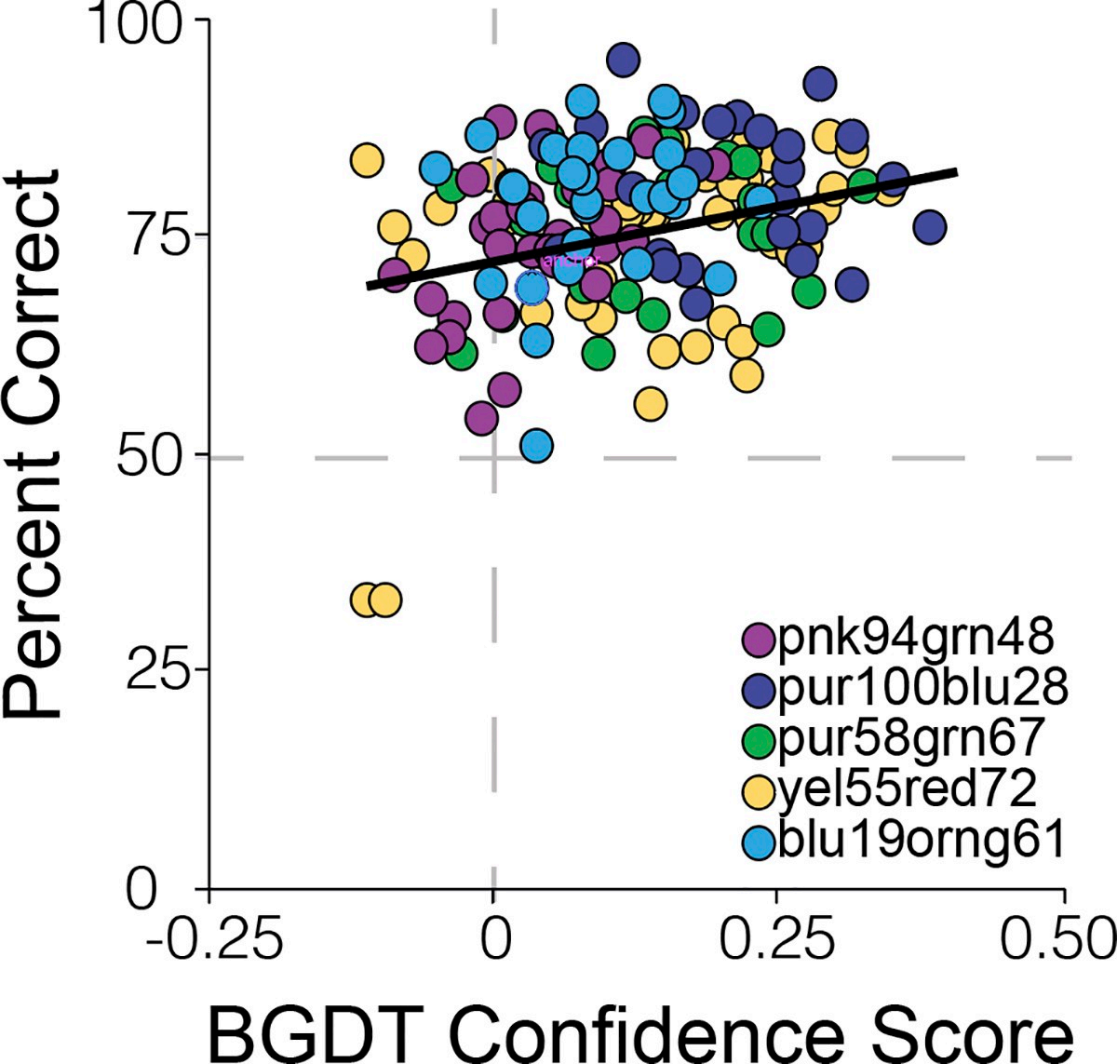
Machine learning algorithm predicts female performance. The confidence score of the combined classifier, quantified as the distance of the BGDT prediction score from the decision threshold (0.5; higher positive values indicate greater confidence and accurate classification, negative values indicate incorrect classification), on probe stimuli significantly predicted the accuracy of female performance on the same stimuli. Colors indicate the male stimulus set.

To gain deeper insight into the similarity in performance of the classification algorithm and female zebra finches, we compared the performance of the algorithm and of females on each individual probe stimulus. Briefly, we asked the combined classifier to categorize each probe stimulus (these exemplars were not used for network training; performance accuracy on probe stimuli from these five males was 89.1±3.8%). We then calculated a “confidence score” as the distance from the decision threshold on each probe stimulus, to reflect how confident and accurate the classifier was in its prediction (see Methods). With this metric, higher positive values indicate greater confidence and accuracy, while negative values indicate incorrect classification. We then tested the extent to which the confidence score of the combined classifier predicted performance of female birds. Broadly speaking, probe stimuli that the classifier incorrectly categorized were also those that females misclassified more often, whereas probe stimuli that the combined classifier correctly categorized were stimuli that females also accurately categorized (mixed effects model: F_(1,155.8)_ = 15.7; p = 0.0001). Furthermore, the confidence score of the classifier significantly and positively predicted the accuracy of female zebra finches in categorizing individual probe stimuli as courtship vs. non-courtship motifs (F_(1,101)_ = 11.34; p = 0.0011; Fig 5). This was also true for the classifier that used only the top 50 HCTSA features for classification (F_(1,105.2)_ = 10.42; p = 0.0017). This suggests that the features weighted in the classification decisions by the algorithm could be comparable to those used by females during this form of social decision-making.

## Discussion

Identifying the features that provide information about performance and social context is fundamental for understanding animal communication. Across a range of species, males adjust features of vocal performance depending on their audience, and changes in acoustic features when males direct songs to females are hypothesized to increase the salience or attractiveness of their songs to females [10,20,28]. Thus, the comparison of courtship and non-courtship song offers an excellent opportunity to discover acoustic features that can be used by female receivers to encode male performance and used for social decision-making. Here we first demonstrated that females can discriminate between brief epochs of courtship and non-courtship song (<1 sec each) that contain similar syllables and syllable sequencing. We then adopted a bottom-up approach to reveal acoustic features of these short motifs that female songbirds could use for social discrimination. In particular, we computed thousands of time-series features of acoustic waveforms of courtship and non-courtship motifs (HCTSA; [25,26,29]) and then trained machine learning algorithms to discriminate between these types of motifs. The algorithm reliably discriminated between courtship and non-courtship motifs, generalized to novel exemplars, and identified clusters of features that could be used for classification. Moreover, the algorithm reliably predicted the discrimination performance of females.

Spectral features of individual syllables have been reported to vary between courtship and non-courtship song (reviewed in [21]) and, consistent with this, our algorithm captured differences in spectral features (e.g., features based on power spectral densities (PSD) using different transformations). For example, the distribution of the PSD was found to be consistently wider for non-courtship song than for courtship song (i.e., higher variance (log iqr) of the PSD; S1 Table). This suggests that males produce a narrower range of sounds when directing songs to females during courtship interactions, despite using the same syllables in courtship and non-courtship motifs. In addition, while the central tendency of the PSD was not among the top 50 features for discrimination, we observed that the medians of the PSDs were generally higher for courtship motifs than for non-courtship motifs. This observation is consistent with previous studies demonstrating that zebra finches produce syllables with flat, harmonic structure at higher fundamental frequencies during courtship singing [30,31]. Consequently, our approach reveals patterns of context-dependent song modulation that are consistent with previous studies.

Importantly, our analyses also revealed novel operations that can be used to differentiate courtship and non-courtship songs. For example, in addition to spectral operations, time irreversibility and entropy operations could be used to classify individual motifs. Time irreversibility is a fundamental feature of non-linear systems and relates to the degree to which processes that describe the forward signal differ from those that describe the time-reversed signal (reversible signals have values closer to zero)[32–34]. The operations in the HCTSA toolbox are based on the assumption of conservation of entropy, as time irreversibility increases when a time series is more entropic across time [35–37]. Time irreversibility has been used to characterize ecological, epidemiological, and engineering time series data [33,38,39], as well as to classify normal and pathologic patterns of neural and cardiac activity and of limb movements [40–45]. However, time irreversibility has not been considered as a metric to describe social behavior, including the structure of communication signals. Inspection of raw and normalized values revealed that the acoustic waveforms of non-courtship motifs were consistently more time irreversible than courtship motifs (S1 Table) and suggest that non-linearities that manifest over time in vocalizations could be an important feature encoded by sensory processing areas.

In general, time irreversibility measures indicate non-linear (non-Gaussian) properties in the signal. Non-linearities are apparent in the vocal signals of a range of species and can be introduced by the vocal periphery [46]. For example, in zebra finches, the isolated syrinx acts as a non-linear dynamical system and shows rapid transitions between distinct oscillatory states [47]. On the sensory side, while feature extraction occurs throughout the auditory system [17], the degree to which neurons encode non-linear features such as irreversibility is unclear. However, a number of regions in the auditory and associative pallium, such as the caudomedial and caudocentral nidopallium (NCM and NCC, respectively) and the caudomedial mesopallium (CMM) differentially respond to courtship and non-courtship songs [22,23,48], and it is possible that neurons in these regions may be sensitive to variation in time irreversibility or other non-linearities in acoustic signals.

Our findings indicate that there is sufficient information in a single motif for females to assess differences in social context and to make social decisions. Previous studies have found that female zebra finches can distinguish between bouts of courtship and non-courtship song and prefer to listen to courtship song bouts. Courtship and non-courtship song bouts differ in broad scale patterns of song structure, including the number of motifs and introductory notes in a bout (reviewed in [21]); for example, whereas courtship song bouts typically contain 5–7 motifs, non-courtship song bouts often consist of only 1–2 motifs [49–52]. Females could discriminate between courtship and non-courtship song bouts using these broad scale features or, given the repeated nature of song motifs in a bout, discern the stereotypy of vocal performance across motifs. However, it had been unclear whether females could use information available in a single motif to distinguish between courtship and non-courtship songs (see also [53]). We found that females were able to consistently and accurately classify motifs, and do so by forming general categories of stimuli and not by memorizing the individual examples.

Taken together, these data highlight the power of expansive time-series phenotyping to reveal acoustic features that could be important for social discrimination and underscore the promise of such approaches for other classification problems. While other studies have also implemented bottom-up approaches to focal questions in animal communication (e.g., [12,54,55]), none have integrated such an expansive extraction of time-series features, and we highlight the potential of this approach to reveal novel features for deciphering and decoding animal communication signals. This exploratory approach could serve as the basis for additional experiments using other types of acoustic stimuli, in other species, and across sexes. Future studies should consider the degree of collinearity between features and integrate methods to manipulate key aspects of stimuli. Finally, by linking the performance of computational algorithms to behavior, our analyses suggest the biological plausibility of these computations and the potential impact of this approach to understanding complex phenotypes.

## Methods

### Ethics statement

Procedures adhered to UCSF and McGill University IACUC approved protocols and National Institutes of Health guidelines for the care and use of animals.

### Animals

Zebra finches (>90 days post-hatch) were housed either at the University of California, San Francisco or McGill University and were kept on a 14:10 light:dark schedule. Females were housed in operant training boxes within sound isolation chambers (“soundboxes”) and earned access to food based on the task (see below). Food was provided *ad libitum* and enrichment (lettuce, egg food) was provide once per week to females when they were not undergoing behavioral training and to males used for song recording. All animals were provided with water *ad libitum*.

### Behavioral task and training

We trained adult female zebra finches (n = 11) to perform a classification task in a two-alternative forced-choice paradigm. Birds were house individually in custom-built operant cages (D. Floyd and K. McGary, University of California at San Francisco) inside soundboxes. The operant cages interfaced with a TDT RX8 board (Tucker Davis Technologies, Alachua, FL) via custom-written MATLAB software (K. Nagel and H. McLendon). Each soundbox contained a single speaker (Bose, Framingham, MA), and playback intensity was calibrated using pure tones and a calibrated microphone (Brüel and Kjaer, Naerum, Denmark).

During operant training and testing, birds earned access to a food hopper by performing the task (water was provided *ad libitum*). To begin a trial, the female hopped onto a central “song perch” that triggered playback of a single song motif from one male zebra finch (Fig 1A). The female then determined whether the motif was a courtship or non-courtship motif, and reported her choice by hopping on one of two response perches. If she classified the song correctly, a feeder located under the song perch was raised for 4–5 s, providing her with temporary access to food. If she classified the song incorrectly, no new trials could be initiated for a time-out period of 30–90 s. Females were not allowed to respond until motif playback was complete. If the bird did not respond within 5 s of the end of the motif, the trial was scored as having no response, and the female could initiate a new trial by hopping again on the song perch.

There were four stages of training. In the “song stage,” food was freely available, and hopping on the song perch led to playback of a song that was drawn at random from a database of 28 song renditions from four different zebra finch males (“habituation database”). Importantly, none of these stimuli were used for training and classification trials. In the “food stage,” birds hopped on the song perch, which triggered playback of a song from the habituation database, or hopped onto one of the two response perches, which raised the feeder for 4–5 s. Food and song could be procured independently. In the “sequence stage,” birds had to hop first on the song perch, and then hop on either of the two side perches within 5 s of hopping on the song perch to receive a food reward. Finally, in the “discrimination stage,” birds began classification trials of courtship and non-courtship song. The habituation song set was replaced with courtship and non-courtship songs from one male, and trials were rewarded (with food) or punished (with a 30–90 s time-out) depending on which response perch the female chose. Birds spent one or two days on each of the first three training stages (song, food, and sequence) and moved to the next stage of training when they performed at least 200 hops per day (for song stage), or earned at least 200 rewards per day (for food and sequence stages).

If females developed a side bias during the song discrimination phase (i.e., consistently chose the perch on one side regardless of the stimulus), we altered the reward rate so that the preferred perch was rewarded at a lower rate (60–85%). Once biases in performance were corrected, reward rates were equalized over the next one to three days. We excluded data from days on which the two perches were rewarded at different rates from further analyses (see [56]).

Females could work continually for the duration of their 14-h day and generally received all of their food by performing this task. Bird weight was monitored closely, and birds received supplemental food if their weight decreased by more than 15% of their baseline weight. After birds reached criterion in their discrimination behavior, we moved to a variable-ratio partial reinforcement schedule to maintain a high response rate and reduce extinction [57–59]. For this, the overall reward rate for correct trials was lowered to 75–95%. Whether a bird was rewarded or not on a given trial in which they answered correctly was determined randomly. Correct trials that did not produce a reward did not produce punishment, but all incorrect trials continued to be punished with a time-out.

### Classification of novel (probe) stimuli

We used probe trials to test the ability of trained females to generalize the learned classification to new stimuli. Probe stimuli were novel motifs from the same male heard in the training set. Probe trials were introduced only after birds reached asymptotic performance and were performing stably at the partial reinforcement reward rate. Probe trials were randomly interspersed between training trials at a low rate (10–20% of trials), rewarded at a fixed rate equal to the overall reward rate (75–95%) regardless of which response perch the female chose, and never punished. This ensured that no information about the “correct” category of a probe stimulus was contained in its reward rate and that females could not easily discriminate probe trials from normal trials on the basis of reward. Probe stimuli were pseudorandomly selected and all stimuli were played before restarting the stimulus list. Probe trials that did not receive a response were not rewarded and remained on the stimulus list until they elicited a response. We considered females that performed above chance for both courtship and non-courtship probe stimuli to have ‘generalized.’

### Song stimuli

We used song bouts from 17 male zebra finches as they sang to females (courtship song) and as they sang alone (non-courtship song). For songs from 12 of the males, motifs were taken from a stimulus set used in a previous study testing female preferences for whole bouts of courtship and non-courtship song [22]. For these 12 males, we extracted all motifs from each courtship and non-courtship song bout (mean: 32 motifs/context; range 6–85 motifs/context).

For an additional five males, we generated stimuli using the first motif of a song bout. We prepended the same two introductory notes before the start of the motif for all stimuli. Thus, for song discrimination, females were limited to the spectral and temporal cues present within a single motif. From the song bouts of each male, we selected 23–29 courtship motifs and an equal number of non-courtship motifs for use as training stimuli and an additional 13–24 courtship and non-courtship motifs (an equal number of each) for use as probe stimuli (Fig 4B). The stimulus amplitudes were set to 73 dB RMS. Probe stimuli for each male were pulled from a random subset of the original recordings. Songs selected for probe stimuli were interspersed between songs selected for training stimuli within a recording session. The overall power spectra were similar for the courtship and non-courtship stimuli for both training and probe stimulus sets.

All songs were recorded as previously described [23,60]. Briefly, males were housed individually in a cage inside a soundbox containing a microphone and a video camera. Sound foam was placed on the floor of the cage to minimize movement-related noise. Vocalizations were recorded using a custom written sound-activated recording system or Sound Analysis Pro (SAP; [27]; 44.1 kHz). To collect courtship song, we placed a cage containing a female next to the male’s cage in the soundbox and observed the male’s behavior on a video monitor. In 12 cases the female stimulus animal was muted, whereas in 5 cases, non-muted females were used as stimulus animals. During the performance of courtship song, males orient toward the female, fluff the body feathers while flattening feathers on top of the head, hop, dance, and beak wipe. Only songs where males performed at least two of the above courtship components were considered to be courtship songs. After removing the female, we waited up to 10 minutes before reintroducing the female in order to collect interleaved bouts of non-courtship song. We also recorded an additional one hour of non-courtship song before the first and after the last female presentation on each recording day. Males were recorded in the morning and were recorded over multiple days to increase the collection of courtship and non-courtship song bouts for stimulus generation.

Only motifs without background noise or movement artifacts were used as stimuli. Stimuli were normalized (mean intensity for all stimuli was 73 dB RMS SPL, calculated over the total duration of the stimulus) and filtered (Butterworth 2-pole 250 and 8000Hz). Distributions of total duration, RMS volume, and overall power spectrum were similar between all stimuli, but subtle differences in syllable structure and timing between courtship and non-courtship song were preserved.

For songs from three of the five males used for the operant conditioning experiments, we removed the recorded sound between syllables to reduce noise between syllables, and cosine-ramped the beginning and end of syllables to prevent harsh onsets and offsets. This did not affect the performance of females or the machine learning algorithm.

### Quantifying learning during operant training

To quantify learning, we first fit a sigmoid curve to a plot of performance accuracy (% correct) across all days in which females performed at least 20 responses. We then calculated the probability of performing above the lower 95% confidence interval of the horizontal asymptote. Using a geometric distribution we determined the number of consecutive times this level would have to be repeated to appear non-random given the number of days in which females performed the task. Learning was defined as the first day of a set of consecutive days above the asymptotic threshold. If the criteria was not reached, birds were considered to have not learned the task for the stimulus set (n = 2 females), and these data were excluded from the analyses. For probe trials, we calculated the percent of correct responses on all days of probe testing. We did not see significant differences between the first and last day of probe trials, which is as expected because responses to probes were not rewarded or punished.

### Feature extraction using HCTSA

The HCTSA toolbox extracts thousands of features from time series data. For the HCTSA feature extraction, each of the motifs in the datasets was represented as a vector ${\bar{x}}^{k_{i}}$ where the subscript and superscripts correspond to the i^th^ motif in k^th^ dataset (i.e., bird) respectively. P_j_ represents the j^th^ operation and its output is a feature vector F_ij_^k^ = P_j_(${\bar{x}}^{k_{i}}$). Therefore, the HCTSA toolbox operates over each motif and provides a feature matrix F_ij_^k^ (belonging to R^mxn^) where m is the number of motifs within the k^th^ dataset and n is the number of features (Fig 3; [25,29]). For example, the HCTSA toolbox extracts features from two operations related to time reversal asymmetry indices (i.e., time irreversibility), CO_trev and DK_trev [26,43,61]. CO_trev computes a normalized nonlinear autocorrelation of a time series data and is computed as:

CO_TREV(τ)=1N−τ∑n=τ+1N(xn−τ−xn)3(1N−τ∑n=τ+1N(xn−τ−xn)2)32

where x_i_ is the time series value at the i^th^ time point, τ is a specified time lag, and N is the length of the time series. In the HCTSA feature computation, this operation is calculated with various time lags (τ; 1–3), including the first zero-crossing of the autocorrelation function and the first minimum of the automutual information function. We modified this equation slightly from the original HCTSA toolbox such that CO_trev and DK_trev values have similar directionality with regard to irreversibility.

DK_trev, another time reversal asymmetry statistic, computes values at a defined time lag (τ) and embedding dimension (M) [26]. It first computes a lag embedding matrix A (of dimension ((N-τ) x M)) for a given time series (where N is the length of the time series), and then it performs an element-wise multiplication. In the HCTSA calculation, M = 3 and the index is computed as:

$$DK\_\mathrm{TRE}V(\tau )=\langle \left(a_{1}\circ a_{1}\circ a_{2}-a_{2}\circ a_{3}\circ a_{3}\right)\rangle$$

Where < > represents the calculation of the average, and a_i_ is the i^th^ column of the matrix A. In the HCTSA toolbox, this feature is computed with time lags of τ = 1,2,3,4.

### Feature extraction using Sound Analysis Pro (SAP)

We used the “Explore and Score function” within SAP2011 (http://soundanalysispro.com/; [27]) to compute 12 default features from each motif (n = 17 birds): the mean pitch and mean amplitude and the mean and variance of the mean frequency, Wiener entropy, pitch goodness, frequency modulation, and amplitude modulation.

### Classifier training

A bagged decision tree (BGDT) classifier was used to classify motifs as belonging to courtship or non-courtship songs. The BGDT classifier was run using MATLAB (Mathworks, Natick, MA) on each individual dataset (i.e., a set of courtship and non-courtship motifs from one bird; “individual classifiers”) as well as on the aggregate dataset in which data from all birds were combined (“combined classifier”; see Results). Before classification procedures, features extracted from HCTSA or SAP were normalized using a robust sigmoidal transformation, which is resistant to outliers [25]. In addition, because songs varied across individual zebra finches and because we wanted the combined classifier to differentiate between courtship and non-courtship motifs and not between individual birds, data were also normalized after aggregating the data for all 17 birds. Data were normalized by subtracting the minimum value for the feature and dividing the data by the maximum value (i.e., data normalized to 0–1).

Bagged decision trees derive their name from a bootstrap algorithm with aggregation. Bootstrapping is process of re-sampling (with replacement) from a large dataset to create different subsets of data. In this case, subsets of randomly chosen features from each motif were created using bootstrapping. A separate decision tree is trained on each randomly bootstrapped sample, and each decision tree outputs a “vote” as to whether a motif is a non-courtship or courtship motif. In the end, the majority vote across all trees is computed for classification. Hence separate decision trees models are trained on slightly different features and the results are averaged together (aggregation). The aggregation of predictions from sets of decision trees typically results in a model with less variance (compared to a single decision tree whose output can be highly variable), less overfitting, and more generalization.

Observations that are not selected for each sample set are called out-of-bag samples, and these can be used as a “test set” for each model. We performed a 10-fold validation of the BGDT model to get a reliable assessment of performance. For this, we used the ‘KFold’ option provided in Matlab’s Classification Tree model. First, we randomized the entire dataset (e.g., all motifs for the combined classifier) and then divided each dataset into 10 equal subsamples. We then trained the classifier on nine of the subsamples (i.e., 90% of the data) and tested the accuracy of the trained algorithm on the remaining subsample (i.e., “test set”; i.e., 10% of the data). We repeated this process 10 times such that each of the 10 sets was used once as the test set. Performance of the algorithm across test sets was computed across the 10 iterations and reported in the manuscript. Matlab provides built-in functionality of this process through the crossval and kFoldPredict functions. Repeated iterations of classification lead to small variation in cross-validation accuracy, and the median accuracy across 10 iterations is reported here.

In addition, BGDT classifiers also provide predictor importance scores for each input variable (i.e., each of the features from the HCTSA extraction), which can be used to identify relevant features for categorization. These importance scores were used to identify features that are most useful for discrimination (Tables 1 and S1 and S2 Fig).

Confidence scores for the classification of probe stimuli were computed and compared with female performance on these stimuli. The BGDT algorithm outputs a score that ranges from 0–1, with values <0.5 leading to a prediction of “non-courtship” and values >0.5 as “courtship”. Thus, the difference of the output from 0.5 reflects the confidence of the algorithm in its estimate, and we labelled this difference as the “confidence score” of the classifier. We also factored in whether the classifier was correct or incorrect in its classification such that positive confidence scores reflect confidence for correct classifications and negative scores reflect confidence for incorrect classifications. Female performance was the average performance (percent correct) of females on the individual probe stimuli.

### Statistical analysis

In general, we used linear mixed effects models that included female ID as a random variable to analyze female performance on motif discrimination. All models were conducted using a restricted maximum likelihood approach with unbounded variance components. To test for changes in performance over time (percent correct), the model included the day of testing (first day vs. first day post-asymptote for training, first day vs. last day for probes), male stimulus set, context (courtship vs. non-courtship) and the interactions as independent variables. We ran separate models for training and probe data sets. To investigate performance differences between courtship and non-courtship stimuli for training and probe stimuli (i.e., average percent correct on all tests after asymptote; see Results), we used a model with context (courtship vs. non-courtship), male stimulus set, and the interaction as independent variables. For comparisons of performance on training versus probe stimuli, we used a model with stimulus type (training vs. probe), male stimulus set, and context (courtship vs. non-courtship) as independent variables, and with percent correct or response times as the dependent variable. To investigate the changes in reaction times over time we used a model with reaction time as the dependent variable and context (courtship vs. non-courtship), male stimulus set, response (correct vs. incorrect), time (before vs. after asymptote), and the interactions as independent variables. We tested for whether distributions were significantly different from chance (0.5) using t-tests (H_0_: mean = 0.5). Finally, to analyze similarities in female responses to various stimulus sets, we calculated the Pearson product-moment correlation between all pairs of females tested on each male stimulus set in the percent correct on each stimulus. There were 38 pairwise tests for females on training stimuli, and we report the number of significant correlations out of the total number of tests. Significance was set to α = 0.05 for all tests. All statistical analyses were conducted using JMP Statistical Processing Software (SAS, Cary, NC, USA) or custom-written Matlab code (Mathworks, Natick, MA).

## Supporting information

S1 Fig Lack of relationship between sample size and accuracy. There was not a significant relationship between sample size (total number of courtship and non-courtship motifs; n = 17 datasets) and performance of either the individual classifiers (A) or of the combined classifier on each of the datasets (B; Pearson’s correlation coefficient; p>0.85 for each).
(TIFF)

S2 Fig Bagged decision tree feature importance. Plot of the normalized feature importance score for the 250 features with the highest importance scores. The feature importance decreases sharply and plateaus within the first 50 features.
(TIFF)

S1 Table Top 50 features based on bagged decision trees of HCTSA features. Features are sorted by feature name and operation type for organization and structure. Also included is the direction of difference in average HCTSA score.
(XLSX)
