# Supplementary figures and images for: Behavioral discrimination and time-series phenotyping of birdsong performance
Source: PLoS Comput Biol. 2021 Apr 8;17(4):e1008820. doi: 10.1371/journal.pcbi.1008820 (PMC8049717; doi:10.1371/journal.pcbi.1008820)

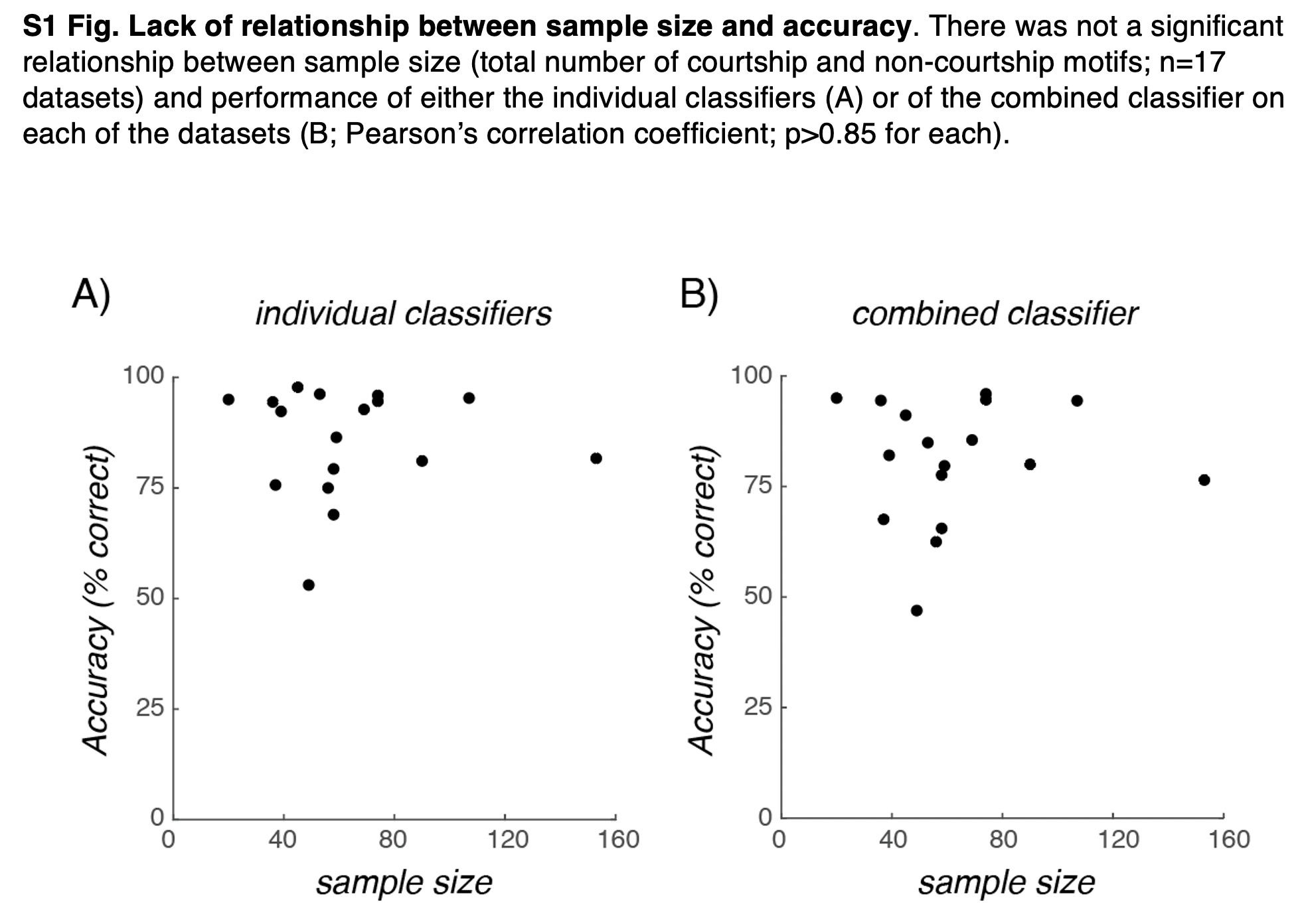

Supplement: S1 Fig — There was not a significant relationship between sample size (total number of courtship and non-courtship motifs; n = 17 datasets) and performance of either the individual classifiers (A) or of the combined classifier on each of the datasets (B; Pearson’s correlation coefficient; p>0.85 for each). (TIFF) [file pcbi.1008820.s001.tiff]

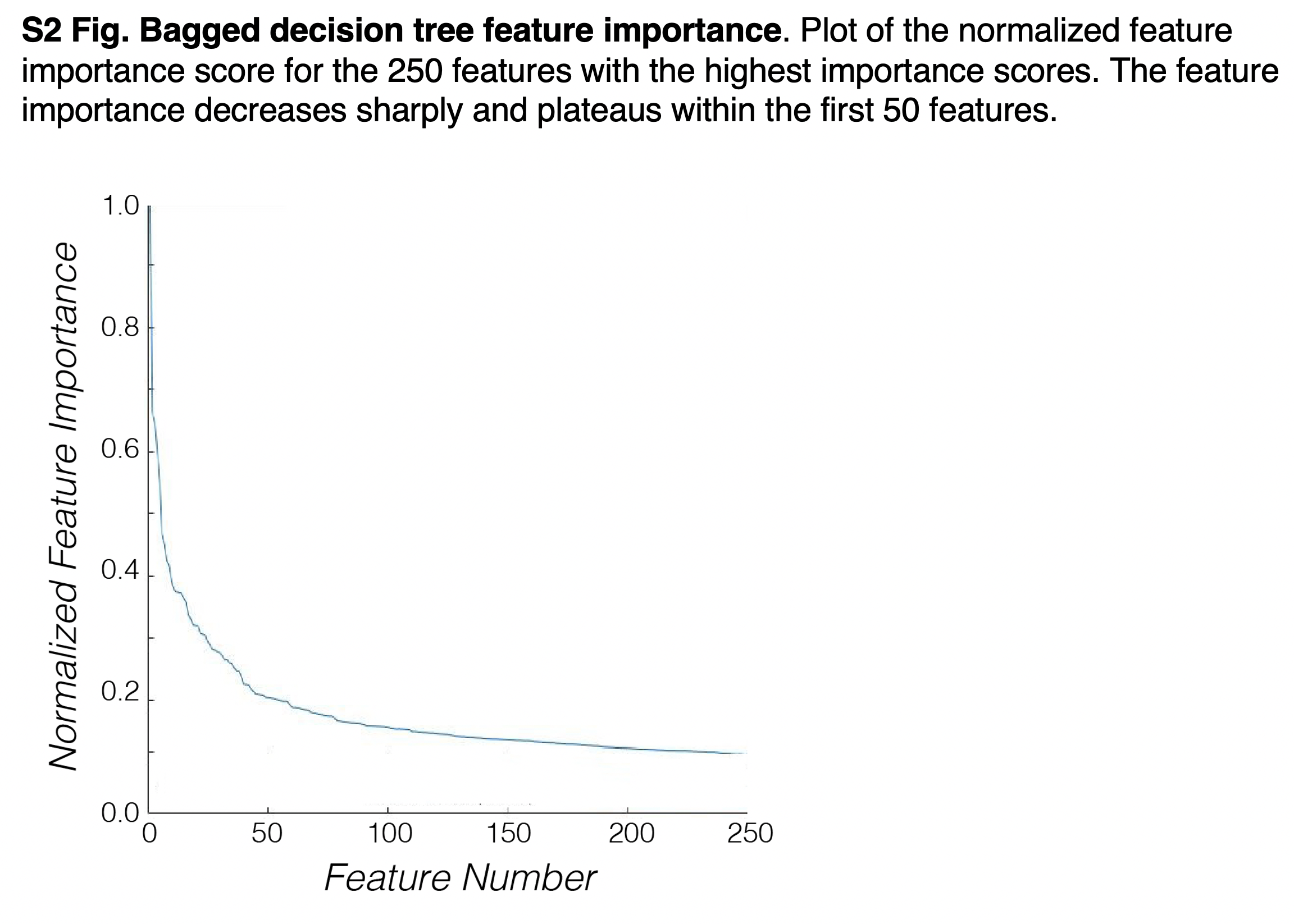

Supplement: S2 Fig — Plot of the normalized feature importance score for the 250 features with the highest importance scores. The feature importance decreases sharply and plateaus within the first 50 features. (TIFF) [file pcbi.1008820.s002.tiff]
